# External validation of nomogram to predict inguinal lymph node metastasis in patients with penile cancer and clinically negative lymph nodes

**DOI:** 10.1590/S1677-5538.IBJU.2018.0756

**Published:** 2019-09-02

**Authors:** Carlos Vaz de Melo Maciel, Roberto Dias Machado, Mariana Andozia Morini, Pablo Aloisio Lima Mattos, Ricardo dos Reis, Rodolfo Borges dos Reis, Gustavo Cardoso Guimarães, Isabela Werneck da Cunha, Eliney Ferreira Faria

**Affiliations:** 1 Departamento de Urologia, Hospital do Câncer de Barretos, Barretos, SP, Brasil;; 2 Associação Piauiense de Combate ao Câncer, Teresina, PI. Brasil;; 3 Fundação Antonio Prudente, A. C. Camargo Cancer Center, São Paulo, SP, Brasil

**Keywords:** Nomograms, Lymph, Lymphatic Metastasis, Penile Neoplasms

## Abstract

**Introduction:**

Penile cancer (PC) occurs less frequently in Europe and in the United States than in South America and parts of Africa. Lymph node (LN) involvement is the most important prognostic factor, and inguinal LN (ILN) dissection can be curative; however, ILN dissection has high morbidity. A nomogram was previously developed based on clinicopathological features of PC to predict ILN metastases. Our objective was to conduct an external validation of the previously developed nomogram based on our population.

**Materials and methods:**

We included men with cN0 ILNs who underwent ILN dissection for penile carcinoma between 2000 and 2014. We performed external validation of the nomogram considering three different external validation methods: k-fold, leave-one-out, and bootstrap. We also analyzed prognostic variables. Performance was quantified in terms of calibration and discrimination (receiver operator characteristic curve). A logistic regression model for positive ILNs was developed based on clinicopathological features of PC.

**Results:**

We analyzed 65 men who underwent ILN dissection (cN0). The mean age was 56.8 years. Of 65 men, 24 (36.9%) presented with positive LNs. A median 21 ILNs were removed. Considering the three different methods used, we concluded that the previously developed nomogram was not suitable for our sample.

**Conclusions:**

In our study, the previously developed nomogram that was applied to our population had low accuracy and low precision for correctly identifying patients with PC who have positive ILNs.

## INTRODUCTION

Penile cancer is less frequent in Europe and in the United States than in other regions of the world. For instance, in South America and parts of Africa, the incidence of PC is high, where it can accounts for 1-2% of malignant diseases (1, 2) in men and represents an important public health issue.

Nodal involvement is the most important prognostic factor (3) in penile cancer, and currently available noninvasive staging methods have low sensitivity for detection of regional lymph node (LN) involvement. Optimal management of patients who are clinically node-negative (cN0) is still debated (4).

Inguinal LN dissection (ILND) can be curative; however, the procedure has high morbidity rates with respect to short- and long-term complications (5). On the other hand, surveillance strategies in patients with cN0 disease (intermediate/high risk, T1b or greater) have been associated with worse survival rates in recent non-randomized, retrospective studies (6-8).

Other alternatives, such as ultrasound-guided fine-needle biopsy, dynamic sentinel node biopsy (DSNB) (9, 10) or minimally invasive approaches, including pure laparoscopic or robotic-assisted ILND (11-14), have been described. However, these methods are dependent on technology, expertise, and have high costs; moreover, their advantages remain unclear.

Nomograms are low cost prediction tools for quantifying individual risk based on prognostic factors, which could be helpful in developing countries. For several cancers, nomograms might provide more precise prediction compared with the traditional tumor-node-metastasis (TNM) classification. Zhu et al. (15) developed a nomogram based on clinicopathological features (T stage, grade, lymphovascular invasion, p53 expression) of penile cancer and clinically negative inguinal LNs (ILNs). This nomogram was designed to predict ILN metastases in squamous cell carcinoma of the penis, to spare patients from unnecessary ILND, especially those living in poor countries. However, the nomogram still requires external validation. The objective of this study was to conduct external validation of the nomogram developed by Zhu et al. (15), based on our population.

## MATERIALS AND METHODS

After receiving Institutional Review Board and ethics committee approval for the study, we included 65 men between 2000 to 2014 who underwent ILND as a part of treatment for primary penile squamous cell carcinoma and who presented with cN0 stage disease preoperatively. The definition of cN0 in our study was nonpalpable ILN. All patients were classified according to the European Association of Urology Risk Classification (EAURC) of penile cancer (16). In our routine practice, we normally suggest bilateral ILND for all patients who are classified as intermediate or high risk, according to the EAURC (17) ILND is generally performed 2-6 weeks after primary disease resection. The time from presentation to primary disease treatment was unavailable because this information was unreliable in the medical records. All pathological reviews were performed by an uropathologist using primary tumor slides. Tumor stage was assigned using the 2002 American Joint Committee on Cancer (TNM) system (18). T2 stage was divided into two subgroups, as in the nomogram by Zhu et al., (15) based on depth of invasion (T2a and T2b, corpus spongiosum and corpus cavernosa involvement, respectively). We used T1a and T1b jointly as category T1 and used Broders system to classify the histologic grade (18) in the same manner as in the nomogram. Lymphovascular invasion and p53 expression (cut-off expression of 20%) (19) were also evaluated in our study. We collected data from patients at three different instituitions, then we performed external validation of the nomogram by Zhu et al. (15).

### Statistical analysis

Data were analyzed using frequency and percentages for qualitative variables and medians and ranges for continuous variables. Comparisons between groups were performed using the chi-square or Fisher’s exact test for qualitative variables and the Mann-Whitney test for quantitative variables. Performance was further quantified in terms of calibration and discrimination. Discrimination was quantified with the area under the receiver operator characteristic (ROC) curve. Calibration was estimated by graphic representation of the associations between observed outcome frequencies and predicted probabilities (calibration curves) for the patient groups. A logistic regression model for positive LNs was developed based on predictor variables: T staging, tumor grade, vascular invasion, and p53 expression. Statistical analyses were performed using two-sided p<0.05 as significant. Models, statistics, and figures were prepared using IBM SPSS software version 23.0 (IBM Corp., Armonk, NY, USA) and R 3.2.21 (http://www.cran.r-project.org).

We considered three different external validation methods for the nomogram by Zhu et al. (15): k-fold, leave-one-out, and bootstrap. We sought to validate and verify whether this nomogram was useful for the prediction of positive ILN with good estimates in terms of confidence intervals.

## RESULTS

This study analyzed 65 men with stage cN0 (intermediate/high risk) penile cancer who underwent ILND for nonpalpable ILN (Table-1) from 3 institutions in Brazil. The mean age was 56.8±14.7 years (range, 25-86 years). Twenty-four (36.9%) patients presented with positive LNs (Table-1) on ILND. Either standard or modified ILND was performed in all patients. Superficial and deep ILN were removed. A median of 21 (range, 3-60) ILN was removed and the mean number of positive ILN was 2.4 (range, 1-12). T1 stage was observed in 16 (24.6%) patients, T2a in 25 (38.5%), T2b in 7 (10.8%), T3 in 16 (24.6%), and T4 in 1 (1.5%) patient. Low-grade tumor (G1) was observed in 20 (30.8%) patients and G2 (61.5%) in 40 patients (Table-1). Comparing stage and ILN metastases, 8/16 (50%) patients with T1 stage, 13/32 (40.6%) with T2 stage, 3/16 (18.8%) with T3 stage, and 0/1 (0%) with T4 stage had ILN metastasis, respectively.

**Table 1:** Clinicopathological characteristics in 65 patientes with penile cancer.

Variables		Mean ± dp (min-max)	
Age (anos)		56.58±14.7 (25-86)	

Variables		N	(%)

T stage	T1	16	(24.6)
	T2a	25	(38.5)
	T2b	7	(10.8)
	T3	16	(24.6)
	T4	1	(1.5)
Grade	G1	20	(30.8)
	G2	40	(61.5)
	G3	5	(7.7)
Lymphovascular invasion	Absente	56	(86.2)
	Present	9	(13.8)
p53 Expression	Weak	32	(49.2)
	strong	33	(50.8)
EAU risk classification	low	0	(0)
	Intermediate	14	(21.5)
	High	51	(78.5)
pN stage	N0	41	(63.1)
	N1	11	(16.9)
	N2	11	(16.9)
	N3	2	(3.1)
Pathologic lymph node status	positive	24	(36.9)
	negative	41	(63.1)

In our study, tumor grade was not associated with LN involvement (p=0.538). Regarding histology, we found 30.8%, 61.5%, and 7.7% of tumors to be G1, G2, and G3, respectively (Table-1). On the other hand, only 4.8% of patients with negative LNs had G3 disease. Lymphovascular invasion was present in 20% of patients with positive LNs and in 10% of patients with negative LNs. In univariate analysis tumor grade and lymphovascular invasion were strongly correlated with LN status (p<0.05). In the multivariate analysis, only T stage was statistically significant (p=0.015; Table-2).

**Table 2:** Univariate and multivariate analysis of clinicopathological factors to predict inguinal lymph node metastasis in 65 patients.

Variables	Univariate analysis		Multivatiate analysis	

	% LNM	p-value	OR (95% CI)	p-Value
**T stage**		**0.154**		**0.015**
T1	50.0			
T2a	32.0		0.341 (0.111-1.049)	0.061
T2b	71.4		2.20 (0.399-12.120)	0.365
T3	17.6		0.075 (0.012-0.462)	0.005
**Grade**		**0.010**		**0.737**
G 1	35.0			
G 2	35.0		0.731 (0.282-1.893)	0.518
G 3	60.0		1.489 (0.145-15.235)	0.737
**Lymphovascular invasion**		**0.002**		**0.071**
Absente	33.9			-
Present	55.6		5.965 (0.857-41.507)	
**p53 Expression**		**0.350**		**0.296**
Weak	31.3			-
Strong	42.4		1.789 (0.602-5.318)	

**Figure 1:**
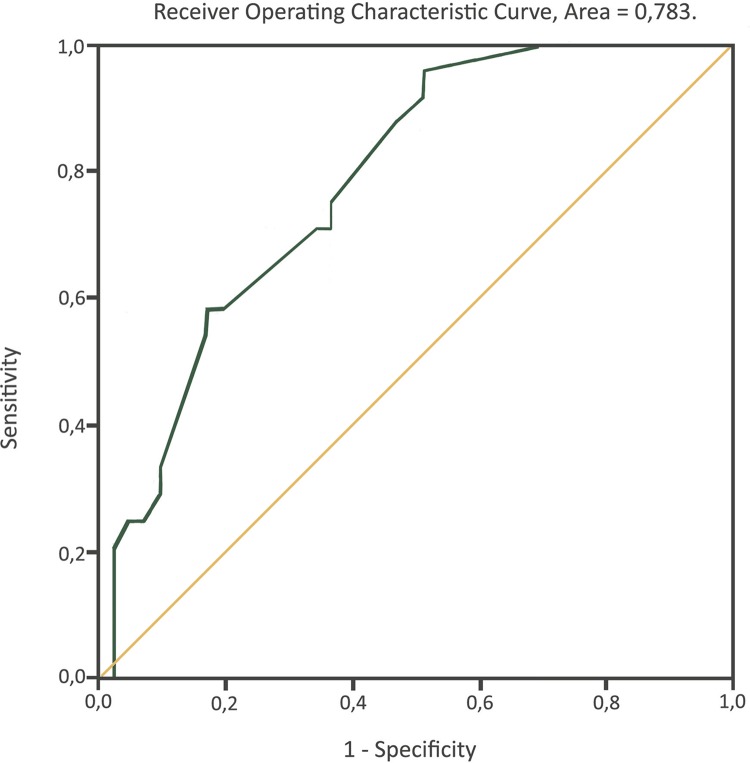
ROC curve generated by Bootstrap method.

Our study included the k-fold, leave-one-out, and bootstrap methods to evaluate the nomogram by Zhu et al. (15). The bootstrap method determined that this nomogram is random and does not establish a pattern of prediction of metastasis. Validation using the k-fold method confirmed this, which we identified during the process of modeling. The predictors shown in the nomogram of Zhu et al. (15) were not statistically significant predictors of ILN metastases in our study sample. All three models showed a low R^2^ (Table-3). These findings demonstrate that the nomogram by Zhu et al. (15) has a high probability of false negatives in our population. The distribution of the bootstrap test results is shown in Figure-1.

**Table 3:** Comparison of results in 3 diferents external validation methods.

Calibration	Zhu’s Nomogram	K-fold	Leave-one-out	Bootstrap
R^2^	0.445	0.228-0.424	0.254-0.389	0.012-0.520
Brier	0.116	0.170-0.195	0.169-0.186	0.141-0.230
**Discrimination**				
(ROC) Area under de curve	0.851	*	*	0.783

* ROC curve was performed for bootstrap only.

## DISCUSSION

There are some nomograms in literature to predict inguinal lymph nodes, for example, one of them was reported by Ficarra et al. (20) and included variables as tumor thickness, grown pattern, grade, LVI, local infiltration, cN stage. Other one was published recently by Peak (21) that used only grade, cN stage, and LVI. Zhu’s nomogram used cT stage, grade, LVI and p53 expression and must be applied in N0 patients. We decided to validate Zhu’s nomogram because of that idea of use a biomarker as p53 expression in association with clinical data, however in our study, this nomogram applied in our population had low accuracy for identifying patients with penile cancer who had positive ILN. Our analysis showed an underestimation of positive LNs. We would like to emphasize that in using the nomogram by Zhu et al. (15) here, we could not improve the selection of patients with positive or negative ILN.

The occurrence and extent of ILN metastasis are the most important prognostic factors in patients with penile cancer and usually imply worse oncologic prognosis (22). Up to 25% of patients with no palpable LNs have occult micrometastases that are not detected by physical examination (23-25), and imaging studies, such as computed tomography scan or conventional magnetic resonance imaging, are also unable to detect inguinal micrometastases (26). Consequently, it could be debated that lymphadenectomy should be performed for all patients with penile cancer (8, 27) because ILN status is the key prognostic factor for survival, and patients can be cured by undergoing ILND. However, this poses a dilemma because early ILND leads to high rates (up to 50%) (28) of complications with significant morbidity, such as infection and/or wound dehiscence, skin necrosis, lymphedema, lymphoceles, and other complications. Surveillance strategies can reduce cancer-specific survival (5, 7-9). Patient survival is over 90% with early lymphadenectomy and less than 40% in patients treated with a surveillance strategy and later lymphadenectomy for regional recurrence. The alternatives, including DSN (9, 10) or minimally invasive approaches such as pure laparoscopic or robotic-assisted ILND (11, 12, 29, 30), are dependent on technology and have high costs, which make them extremely difficult to use in underprivileged populations. Nomograms could be a very interesting tools for improving patient outcome, however in daily practice they are underutilized because the guidelines recommendation of ILND for intermediate- and high-risk tumors (16), other alternatives as DSN and also because of lack of external validation of the available monograms.

Our univariate analysis found that tumor grade and lymphovascular invasion had a strong correlation with LN status. In the multivariate analysis, only T stage was statistically significant. Lymphovascular invasion was the only statistically significant variable in the study published by Zhu et al. (15) whereas we did not find statistical significance for this variable in our study (p=0.212). In patients with positive LNs, 20.8% had lymphovascular invasion; this pathological finding was present in 9.7% of patients with negative LNs. The lymphovascular invasion is a strong predictor of positive inguinal lymph nodes as showed in other studies by Ficarra et al. (31), and other nomogram developed using the National Cancer Database that included 1,636 men in their analysis (21). Our hypothesis is that we found significance only in univariate because of the limited sample.

Zhu et al. (15) developed their nomogram because of the unreliability of currently available modalities for detecting occult nodal involvement, the need for decisive management of regional LNs for improvement of long-term patient survival, and the challenge of avoiding overtreatment with potential treatment-related morbidity. We sought to validate this nomogram for the prediction and identification of patients at risk for nodal metastasis who could potentially be spared unnecessary ILND. In this nomogram, surveillance is recommended if the nomogram probability of positive nodes is 0.1 (10%). The nomogram represents an attempt to define an objective, systematic, standardized, multivariate model capable of providing individual pN stage predictions. In our study, we performed ILND for cases with intermediate and high risk, according to EAU guidelines. Using this classification, we performed 41 unnecessary ILND and detected 24 cases of ILN metastasis. Considering the threshold of 10% prediction risk (Zhu et al.) in our study, we had 35 patients (62%) that underwent ILND unnecessarily (true negatives), and we would have missed 3 (12%) patients with LN metastasis (true positives). Using a threshold of 20%, 31 (59%) underwent ILND unnecessarily (true negatives) and we would have missed the same 3 (12%) patients with LN metastases (true positives).

Despite the fact that this nomogram is a noninvasive and low-cost approach, it requires external validation. The aim of the present study was to externally validate a predictive model for ILN metastasis in our cohort of patients who had undergone ILND. Only pN status performed adequately within our external cohort of patients, and this finding was consistent using different statistical means (i.e., overall performance, discrimination, calibration, and clinical usefulness).

The nomogram proposed by Zhu et al. (15) is basically a model that can be used to explain the variability of one or more variables and the association and correlation of this variability with other exploratory variables. The goal is to determine values for the parameters in the specified template that generate the best fit of the model to the data. The best model is the one that produces the least unexplained variability, subject to the restriction that all model parameters must be statistically significant. One of the most important principles concerning the process of modeling is simplification of the model. The principle of parsimony says that given a set of equally good possible explanations, the correct explanation is the simplest one. Accordingly, given a set of valid models, the best model is the one that: a) includes the least number of variables, B) is linear and contrasts with nonlinear models, C) is based on few statements, and D) recognizes that simple explanations are always preferable in comparison with complex explanations. In the case of the model proposed by Zhu et al. (15), only lymphovascular invasion was identified as a statistically significant predictor for positive ILN. We used the bootstrap method because this method is used to estimate the confidence interval of parameters. In the bootstrap method, we set the answer and performed resampling of predictors (1,000 times) to identify confidence intervals for the parameters of the logistic regression and to identify better and greater values for R^2^, the c-index statistic, and Brier score. Using the k-fold validation method, we measured the accuracy of the model, i.e., the model’s ability to faithfully represent the sample data. We used a third-party validation method, the leave-one-out method, which is a generalization of the k-fold method, where the number of templates is equal to the size of the sample. The method is useful for evaluating the complete behavior of the model and for correcting defects of the model. Considering that, we identified the extremes of confidence intervals for the parameters of logistic regression. Again, we identified the values of R^2^ statistics, the c-index, and Brier score. These analyses confirmed that in our sample, the model proposed by Zhu et al. (15) was inappropriate, and even cross-validation did not improve the model. In our sample, the predictors shown in the nomogram of Zhu et al. (15) were not statistically significant predictors of ILN. All models showed a low R^2^, including with the bootstrap technique (between 0.228 and 0.424) and leave-one-out (between 0.254 and 0.389) method. In the bootstrap method, p53 expression was identified as a better parameter.

We found that accuracy of this nomogram was lower in our sample (area under the ROC curve, 0.79). The calibration plot showed underestimation of positive ILN. This indicates poor sensitivity, poor specificity, and a low positive likelihood ratio for the various values used in the nomogram by Zhu et al. (15). According to our findings, we would like to highlight that the nomogram by those authors does not have satisfactory performance in improving selection of patients with positive or negative ILN disease, even using a threshold of 10% or 20%. The applicability of models derived from cohorts in China may be questionable when transferred to Latin America. These results could be explained for some reasons: different population and race, low accuracy of Zhu’s nomogram, limited sample, lack of other biomarkers, etc.

The limitations of the present study are inherent to any retrospective series. The number of patients was small (N=65); however, considering the rarity of penile cancer, our sample size is similar to those in other published series in the literature. Our population was significant and sufficient for validation of the nomogram by Zhu et al. (N=110) in penile cancer. Lymphadenectomy templates were not standardized; however, the three institutions and the surgeons involved are experts in urologic oncology and have extensive experience in the management of penile cancer. Nevertheless, our data reflect a real-world, multicenter experience.

## CONCLUSIONS

In our study, the nomogram by Zhu et al. (15) applied in our population had low accuracy and low precision for correctly identifying patients with penile cancer who have positive ILN. Our analysis showed an underestimation of positive LNs. Using this nomogram, we could not improve the selection of patients with positive versus negative ILN.
